# Combined Electron Microscopy Approaches for Arterial Glycocalyx Visualization

**DOI:** 10.3389/fcvm.2022.840689

**Published:** 2022-03-09

**Authors:** Laurence Chevalier, Jean Selim, Celia Castro, Fabien Cuvilly, Jean-Marc Baste, Vincent Richard, Philippe Pareige, Jeremy Bellien

**Affiliations:** ^1^Université Rouen Normandie, CNRS, INSA Rouen Normandie- Normandie Université- GPM-UMR 6634, Rouen, France; ^2^Université Rouen Normandie, INSERM, Normandie Université, ENVI- U1096, Rouen, France; ^3^Rouen University Hospital, Department of Anaesthesia and Critical Care, Rouen, France; ^4^Rouen University Hospital, Department of Thoracic Surgery, Rouen, France; ^5^Rouen University Hospital, Department of Pharmacology, Rouen, France

**Keywords:** endothelial glycocalyx, FIBSEM tomography, scanning transmission electron microscopy, scanning electron microsopy, ischemia-reperfusion, pulmonary artery, correlative microscopy

## Abstract

Mainly constituted of glycosaminoglycans and proteoglycans, the glycocalyx is anchored in the plasma membrane, covering, in particular, the extracellular face of the arterial endothelium. Due to its complex three-dimensional (3D) architecture, the glycocalyx interacts with a wide variety of proteins, contributing to vascular permeability, the flow of mechanotransduction, and the modulation of local inflammatory processes. Alterations of glycocalyx structure mediate the endothelial dysfunction and contribute to the aggravation of peripheral vascular diseases. Therefore, the exploration of its ultrastructure becomes a priority to evaluate the degree of injury under physiopathological conditions and to assess the impact of therapeutic approaches. The objective of this study was to develop innovative approaches in electron microscopy to visualize the glycocalyx at the subcellular scale. Intravenous perfusion on rats with a fixing solution containing aldehyde fixatives enriched with lanthanum ions was performed to prepare arterial samples. The addition of lanthanum nitrate in the fixing solution allowed the enhancement of the staining of the glycocalyx for transmission electron microscopy (TEM) and to detect elastic and inelastic scattered electrons, providing complementary qualitative information. The strength of scanning electron microscopy (SEM) was used on resin-embedded serial sections, allowing rapid and efficient large field imaging and previous correlative TEM observations for ultrastructural fine details. To demonstrate the dynamic feature of the glycocalyx, 3D tomography was provided by dual-beam focus-ion-beam-SEM (FIB-SEM). These approaches allowed us to visualize and characterize the ultrastructure of the pulmonary artery glycocalyx under physiological conditions and in a rat pulmonary ischemia-reperfusion model, known to induce endothelial dysfunction. This study demonstrates the feasibility of combined SEM, TEM, and FIB-SEM tomography approaches on the same sample as the multiscale visualization and the identification of structural indicators of arterial endothelial glycocalyx integrity.

## Introduction

In recent years, the endothelial glycocalyx gained interest since many studies proved its fundamental role in the maintenance of vascular physiology and endothelial response to pathophysiological events. Composed mainly of glycosaminoglycans (GAGs), proteoglycans, and glycoproteins, glycocalyx appears as an ultrafine dynamic network covering the luminal side of the vascular endothelium. Major proteoglycan family components connect tightly the glycocalyx to the cell membrane, i.e., syndecan, a membrane-spanning domain, and glypican, a glycosylphosphatidylinositol anchor (1). Others proteoglycans such as mimecan, perlecan, or biglycan are secreted directly after their synthesis and constitute the pool of soluble proteoglycans. The protein core is covalently linked to GAG chains of repeating disaccharide units that differ from each other in their sugar composition; the most abundant is heparan sulfate (HS) composed of glucuronic acid/N-acetyl glucosamine as the primary structure, which then undergoes post-Golgi modifications such as deacetylation or sulfation. The pattern of sulfated or non-sulfated blocks reveals specific binding sites for various proteins along the linear chains of polysaccharides and contributes to several physiological functions of the glycocalyx (2, 3). Nowadays, it is well assumed that alterations of glycocalyx structure mediate endothelial dysfunction and contribute to the aggravation of peripheral vascular diseases. There is also a large challenge to assess glycocalyx ultrastructural modifications under physio/pathological conditions, in particular using imaging techniques. Combining high spatial resolution and analytical tools, electron microscopy (EM) remains the most reliable approach to investigate glycocalyx structure and its cellular environment. The first imaging EM of the glycocalyx was based on sugar reactivity with dye Alcian blue, Ruthenium red (4, 5) or on the interaction of the negatively charged surface with cationic colloids such as dysprosium (6), or combined Lanthanum–Dysprosium in the LaDyGAGa method (7). With the improvement of scanning and transmission electron microscopy SEM and (S)TEM, it became possible to reach multiscale and chemical information, offering a new way to explore the glycocalyx structure. The objective of this study was to propose innovative approaches in EM to visualize the vascular glycocalyx at the subcellular level and to use these approaches to assess the impact of a pathological condition known to profoundly alter vascular function and structure, i.e., ischemia-reperfusion (IR) associated with cardiopulmonary bypass (CPB) which go along with lung transplantation procedures. For this purpose, we develop a workflow based on a single sample preparation protocol applied to SEM observations, (S)TEM chemical analysis, and tomography FIB3D. Pulmonary arteries from male rats were perfused with lanthanum cations, providing a chemical contrast by reacting with sulfated/anionic groups of the glycocalyx. Lanthanum nitrate as glycocalyx labeling allowed to enhance the staining of the glycocalyx for conventional bright-field TEM observations, but also to detect elastic and inelastic scattered electrons, providing complementary information about elemental distribution at a subcellular level. As a first step, we applied the strength of the current SEM on resin-embedded serial sections, allowing rapid and efficient large field imaging of the sample. This approach is particularly well adapted to obtain an overview of a large vascular vessel or to identify a specific region of interest which will then be imaged at higher magnification by (S)TEM observations for ultrastructural fine details. The analytical configurations of today's transmission electron microscope allow for the acquisition of chemical data through the optimization of their detection setup and spectrometer performance as illustrated by the development of the electron energy loss spectroscopy (EELS) on the biological sample (8). Electron energy loss spectrometer collects beam electrons that have lost kinetic energy after their interaction with the sample. The energy losses are directly related to the elemental chemical nature of the impacted atoms and provide quantitative elemental maps, accessible by energy filtered (EFTEM) or STEM-EELS imaging. Finally, to demonstrate the dynamic feature of the glycocalyx, we provided 3D tomography by dual-beam focus-ion-beam-SEM (FIB-SEM) based on the principle of “slice and view.” This study presents the feasibility of combined SEM, TEM, and FIB-SEM tomography approaches for visualizing the vascular glycocalyx on the same artery sample.

## Materials and Methods

### Animals

The experiments were performed on male Wistar rats weighing 400–500 g obtained from Janvier Labs (Saint-Berthevin, France). Animal housing and experiments were in accordance with the National Institute of Health guidelines and the study was ethically approved by the Normandy University Regional Review Board according to French and EU legislation (no. APAFIS 2016102718162386-V2). These animals were issued from the study of 9. This study was realized to investigate the place of the use of routine CPB during lung transplantation. This question remains controversial in the literature. Thus, the objective of this work was to investigate the impact of CPB on pulmonary endothelial dysfunction and glycocalyx degradation in a model of pulmonary IR in rats. The results obtained in this study showed that CPB significantly increased the effects of pulmonary IR on pulmonary vascular dysfunction and glycocalyx degradation.

**Figure 1:**
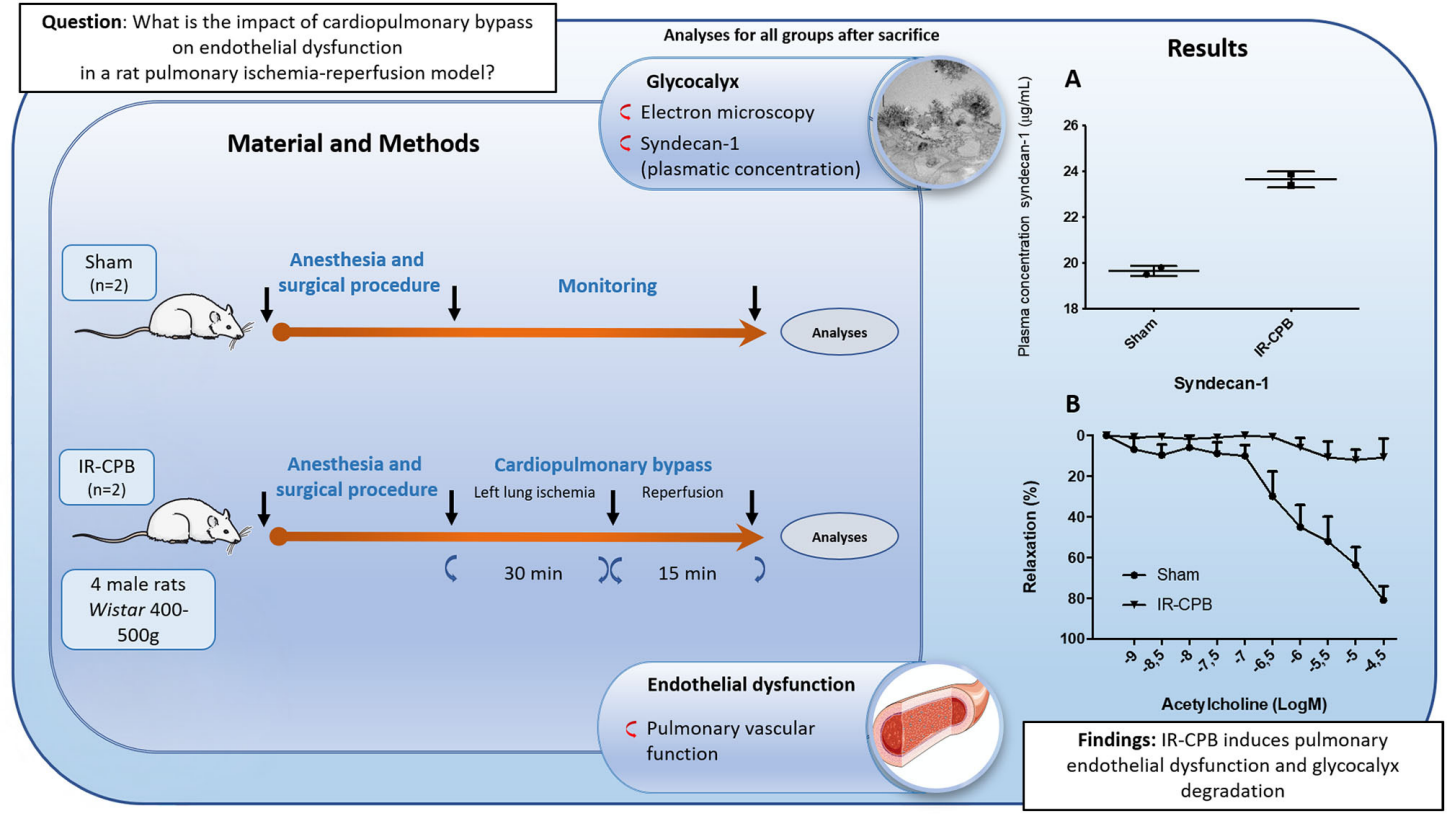
Schematic diagram of the experimental procedure. CPB, cardiopulmonary bypass; IR, ischemia-reperfusion; **(A)** Glycocalyx degradation. Plasma concentration of syndecan-1. **(B)** Relaxation to acetylcholine after precontraction with phenylephrine (endothelium-dependent relaxation). Responses are expressed as percentage relaxation of phenylephrine-induced precontraction (mean ± SEM).

For TEM investigations, analyses were performed on the 2 groups from this study: the sham group (*n* = 2) and a group combining pulmonary IR and CPB (IR-CPB group) (*n* = 2). Regarding the vascular response, we found the same results in these 2 groups as in the study previously described by Selim et al. (9). Data showed increased plasma levels of the glycocalyx marker syndecan-1 in the IR-CPB group compared to the sham group (IR-CPB 23.6 ± 0.3 μg/ml; sham 19.6 ± 0.2 μg/ml). Also, pulmonary endothelium-dependent relaxation to acetylcholine was markedly reduced in the IR-CPB group (10.7 ± 9.1%) compared to the sham group (70.1 ± 8.4%) (Figure 1). In these 2 groups, rats were anesthetized by an intraperitoneal injection of xylazine (10 mg/kg) and ketamine (80 mg/kg). The right femoral vein was cannulated with a 16-gauge catheter (Introcan Safety, BBraun Medical) to administer heparin (500 IU/kg) solution before an infusion of fixative solutions as described below.

### Electron Microscopy Sample Preparation

**Figure 2:**
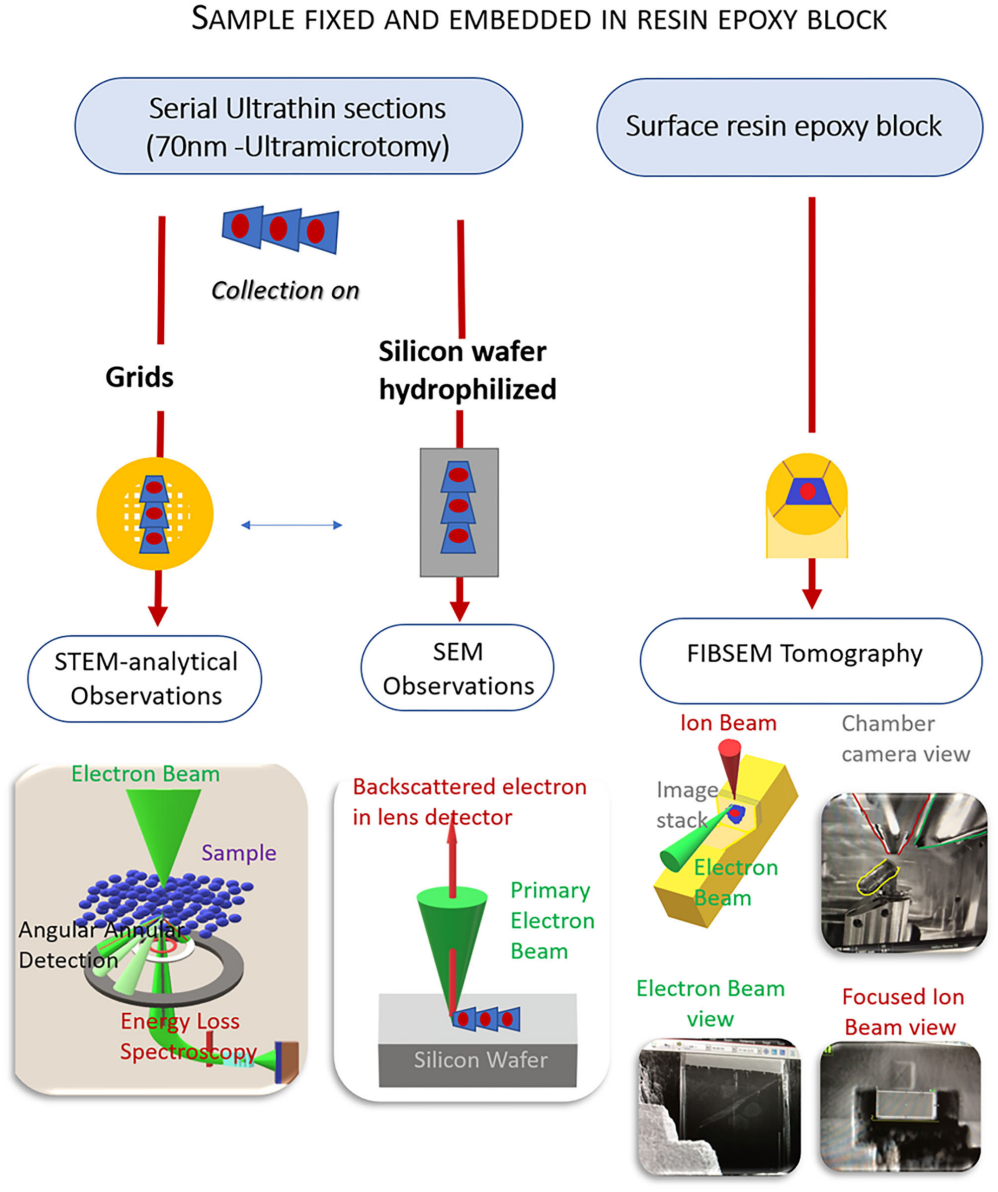
Methodology workflow of electron microscopy (EM) combined approaches from a unique sample. EM preparation follows a standard protocol. From the same sample, ultrathin sections are collected either on grids for scanning and transmission electron microscopy [(S)TEM] analytical observations or on silicon wafers for scanning electron microscopy (SEM) observations. Tomography focus-ion-beam-SEM (FIB-SEM) is performed on the resin block whose surface was smoothed by the previous ultramicrotomy process. The resulting data gives information on the location and distribution of the endothelial glycocalyx in a volume fraction of the artery.

The innovative character of this study is to combine different EM techniques on a single sample prepared under conventional procedures known as *ex-situ* correlative technics. SEM and TEM observations were done on ultrafine sections, while FIB-SEM tomography was prepared from the face resin block (Figure 2).

Rats were perfused through their femoral vein with 5 ml of fixatives containing 2% glutaraldehyde EM grade (EMS), supplemented with 2% nitrate lanthanum hexahydrate (Fluka) in cacodylate buffer 0.1 M pH 7.2 + 2% sucrose. The lungs were exteriorized and dissected out under a stereomicroscope to isolate the pulmonary artery and to obtain small cross-sections (four per rat). During the dissection steps, tissues were kept immersed in fixative solutions to preserve them from collapsing or degradation by air contact. Tissues were fixed again for 1 h at 4°C in the same fixatives solution previously used for perfusion. They were subsequently rinsed in buffer without glutaraldehyde and postfixed 1 h at +4°C with 2% osmium tetroxide (EMS). After a brief rinsing, sample dehydration is performed with cold graded series of ethanol, ended with anhydrous acetone. Infiltration was done in pure low viscosity resin (EMS) under vacuum and polymerization was processed at 60°C for 48 h.

Ultrathin section (70 nm) were cut using a UC 7 ultramicrotome (Leica-microsystems-Vienna) and placed on silicon wafer for SEM observations or 600 mesh gold grids for TEM analyses.

### Transmission Electron Microscopy Observations

Routine TEM observations were performed at 120 kV on JEOL JEM-2010 in bright field mode.

Analytical and high-resolution TEM imaging were performed at 80 kV on a JEOL JEM ARM200F setting with a field emission gun (FEG) and a probe Cs aberration corrector. This TEM is equipped with an energy electron loss spectrometer (GIF-Quantum ER—Gatan-Ametek, USA) upgraded with the Dual EELS option allowing the simultaneous acquisition of two-electron spectra at desired energy. This microscope allowed us to acquire images in scanning-transmission mode, using a high angle annular dark field detector (STEM-HAADF). Based on the electron-matter interaction, electrons submitted to high elastic scattering are collected by an annular detector, resulting in dark-field images and contrast being dependent on the atomic number of collided atoms. Therefore, heavy chemical species appeared brighter than light ones (10). Conditions for STEM acquisition used an 8C probe size, 8 cm length camera, and 50 μm condenser lens aperture diameter.

### Electron Energy Loss Spectroscopy and EFTEM Experimental Conditions

Chemical maps were obtained either by EELS spectrum imaging or by energy-filtered imaging (EFTEM). EELS experiments were performed with a primary voltage of 80 kV and a beam current of 12 pA/cm^2^. Spectra were collected with 6C probe size, 3 cm length camera, 50 μm condenser lens aperture diameter, and 2.5 mm as diameter GIF entrance. Spectra were collected from 0.10 μm × 0.22 μm sample area, with energy dispersion of 0.4 eV/ch and 34 frames were summed. Relative quantification of La (% atomic) was determined based on La N edge (99 eV), C _k_, N _k_, O _k_ core edges. EFTEM La-maps were obtained by the conventional 3-windows method, with an energy slit width of 40 eV. In short, background images acquired in the pre-ionization edge of La-M_5_, M_4_ (respectively 768–808 eV) were computed to remove the background contained in the post-edge image (852 eV), the result leading to the La-map, where the signal is proportional to the element concentration in the sample.

### Scanning Electron Microscopy Observations on Ultrafine Sections

Serial ultrafine sections (70 nm) were collected on silicon wafer previously hydrophilized by plasma AR/O_2_ (RF50W, Ar 35.0 sccm, O_2_ 11.5 sccm) for 4 min (Plasma Cleaner-GATAN-Ametek-USA). The sections were stained for 1 min with a uranyl-less solution (Delta microscopy-France) containing high heavy lanthanum and gadolinium salts, and then were platinum-coated (1 nm) with a precision etching and coating system (PECS-Gatan-Ametek-USA).

Electron micrographs were acquired on SEM JEOL 7,900 F using the gentle super high-resolution stage bias mode. This function improves high resolution at any accelerating voltage and is particularly well adapted to an insulator biological sample. This mode decelerates the illuminated electron beam and accelerates the electron signal using a biased voltage for the sample (HT 7 kV, probe 2.4 nA, WD 3 mm). Images were obtained with the high ultrasensitive backscattered detector improving Z-contrast at low accelerating voltage.

### Tomography FIBSEM

The principle of three-dimensional (3D) imaging is based on the “slice and view” method using a focused ion beam to create a cut at a designated site in the specimen, followed by viewing the newly generated surface with a scanning electron beam. The repetition of these two steps several times leads to the formation of an image stack that can be reconstructed in a representative volume of the sample. Experiments were performed on a xenon plasma FIB-G4 Helios microscope (Thermo Fisher Scientific—Eindhoven). The resin-block, previously smoothed at its surface by ultrathin sectioning for TEM observations, was coated by 200 nm of gold and placed in the SEM on the pretilted stub. A first cross-sectional cut was made at a high ion beam current of 60 pA to generate an approximate 6 μm wide trench, after having protected the exposed surface by a platinum layer. The next slices were cut at 15 nA, 30 kV with a step size of 10 nm (Sham-sample) and 50 nm (IR-CPB sample). SEM images of each slice were acquired at 3 kV, 0.8 nA with the through lens detector collecting backscattered electrons (AutoSlice and View Software Thermo Fisher Scientific—Eindhoven). Stack images were posttreated with Fiji software (11). Noise removal, linear stack alignment, and volume reconstruction were performed with plugins variational stationary noise remover (VSNR) (12), registration (13), and volume viewer (14). To quantify the changes of glycocalyx density in the IR experiment on the 3D FIB-SEM tomogram, we extrapolated the Cavalieri method to define, respectively, the volume fraction of glycocalyx and endothelium. We consider FIB-SEM slices as parallel series of cutting planes at a fixed distance. The volume of the glycocalyx (V_gcx_) and the endothelium (V_end_) are estimated by summing the areas (A) of each object on serial sections and multiplying by the step size (T).


$$\begin{array}{l} V\left(x\right)=\left(TxA1\right)+\left(TxA2\right)+\left(TxA3\right)+\left(TxA4\right)\ldots \ldots \ldots \\ \;=T\;x\;\left(\overset{n}{\underset{i=1}{\sum }}Ai\right) \end{array}$$

We normalized the volume of glycocalyx by the volume of the endothelium, and calculate the volume ratio (%) as $\frac{V\left(gcx\right)}{V\left(endo\right)}$x100. Data are represented in **Figure 6**; the Bar graph shows the glycocalyx volume ratio when the whole 3D tomogram is considered, and blue dots illustrate the dispersion of the glycocalyx volume ratio for each FIB-SEM section respectively for IR-CPB and sham (*n* = 31 and 40).

Areas of interest region were outlined by threshold using Fiji software when contrast is sufficient or manually (11).

## Results

### Contribution of Combined EM Multiapproaches for Glycocalyx Visualization

**Figure 3:**
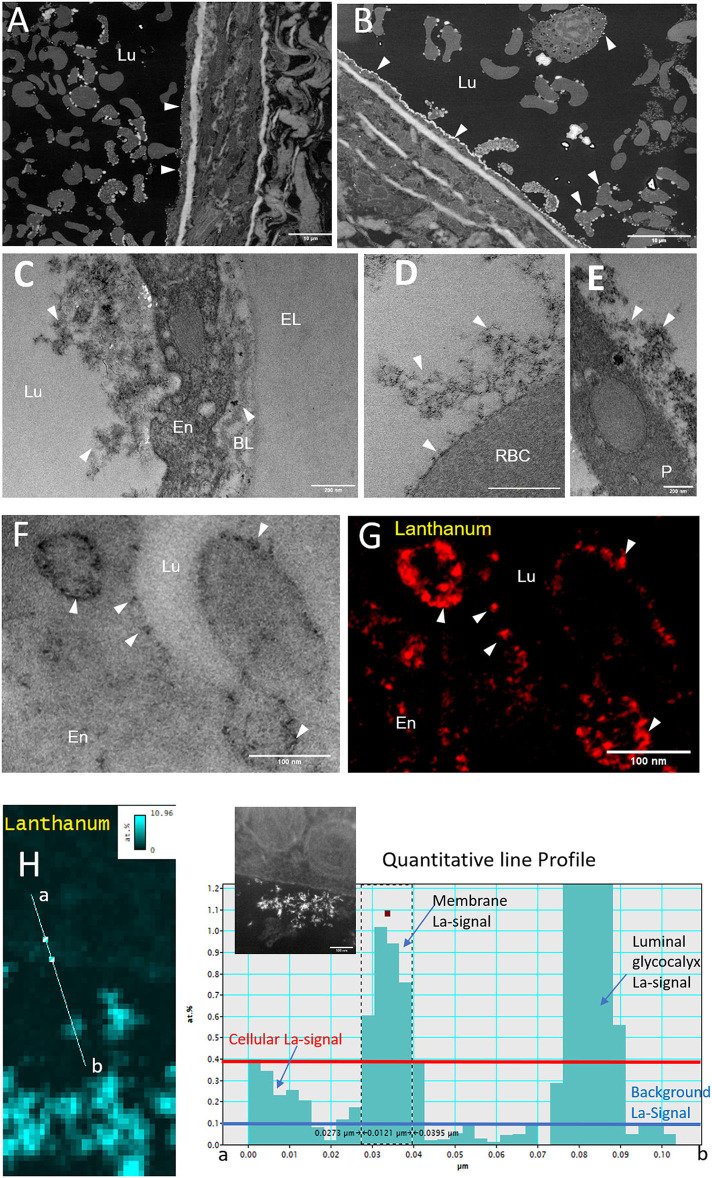
Morphology of the glycocalyx under physiological conditions imaged by EM combined techniques. **(A,B)** Overview micrographs acquired by SEM observations on resin embedded ultrathin sections. Glycocalyx linked to lanthanum is visualized by high brightness contrast at the surface of the endothelium (arrows) and of circulating blood cells (scale bar = 10 μm). **(C–E)** Higher Resolution transmission electron microscopy (TEM) images showing a dense glycocalyx developed at the endothelium, red blood, and platelet surface (scale bar = 200 nm). **(G,H)** Distribution of lanthanum salt by electron energy loss spectroscopy (scale bar = 100 nm); **(F)** Bright-Field Image; **(G)** EFTEM image; **(H)** STEM-electron energy loss spectroscopy (EELS) quantitative La-map (%at.) with line profile. The insert black and white image corresponds to scanning-transmission mode, using a high angle annular darkfield detector (STEM-HAADF) endothelium view (scale bar = 100 nm). BL, Basal Lamina; En, Endothelium; Lu, Lumen; EL, Elastic limiting; P, Platelet; RBC, Red Blood Cell.

**Figure 4:**
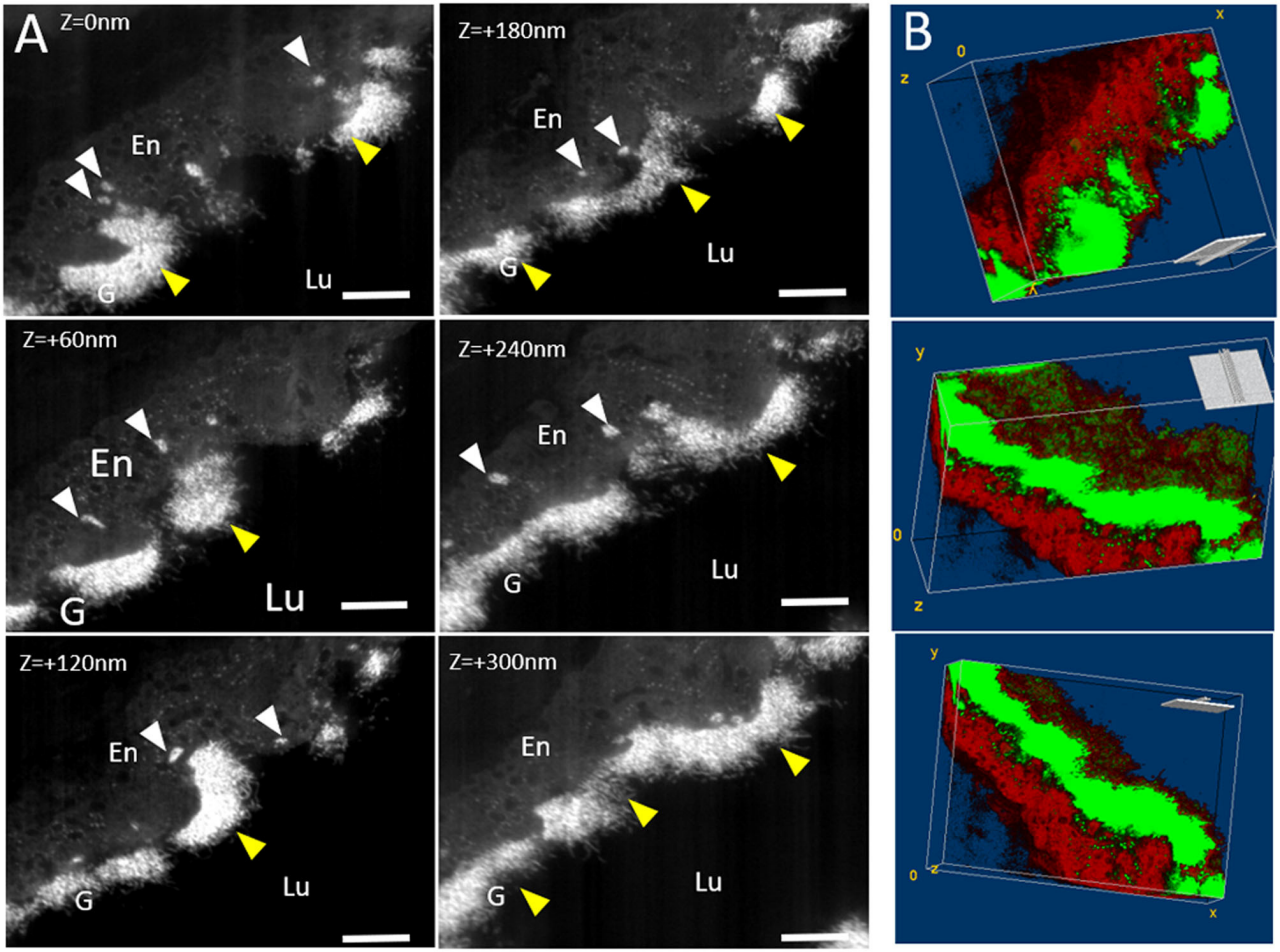
Tomography 3D FIB-SEM under physiological conditions: **(A)** Micrographs extracted from FIB-SEM stack at different z-position. Stack images were acquired from 37 “slice and view” images with a step of 10 nm. Glycocalyx (G) (yellow arrows) appears as white dense-packed material at the endothelial (En). White arrows point endothelial vesicles filled with glycocalyx. **(B)** Volume reconstruction: Glycocalyx is thresholded as green part while the endothelium is in red. Note the presence of glycocalyx inside the intracytoplasmic vesicles. Scale bar = 1 μm.

The visualization of arterial glycocalyx remains a real challenge due to its high sensitivity to the blood flow or the artifact induced by EM preparation. In particular, one major difficulty in TEM study is obtaining a good representation of the native state of glycocalyx morphology, especially in large vascular vessels. A combined EM approach might be helpful to reach this goal. Figure 3 illustrates glycocalyx under physiological conditions. SEM observations of resin-embedded ultrathin sections allowed us to defocus the field of view and to appreciate the global vascular environment of the endothelium as shown in Figures 3A,B (luminal space, circulating blood cells). The glycocalyx labeled by lanthanum cations appears with brighter and higher contrast than the biological matrix and is regularly distributed at the luminal side of the endothelium. The arterial lumen contains red blood cells and immune circulating cells; some of them interact with the endothelium and present a dense-packed white signal on their surface. The free detached glycocalyx is also observed in the lumen in low abundance. Higher resolution images are provided by TEM observations (Figures 3C–E) supporting a well-developed filamentous glycocalyx emerging from the endothelium to the luminal arterial space, but also present on red blood cells (Figure 3D) and platelet surfaces (Figure 3E). In addition, we noticed lanthanum reactivity with the components of the basal lamina. In some cases, the glycocalyx is highly cleaved with only the fraction anchored in the plasma membrane visible. In such conditions, the energy-filtered imaging, based on the loss of energy of incident electrons after their interaction with lanthanum ions, can improve the detection of the glycocalyx compared to conventional bright-field TEM (Figure 3F). Figure 3G refers to the elemental La M_5_ M_4_ map exhibiting high content of La located in endocytic vesicles and sub-domain of the plasmalemma. Another example consisting of STEM-EELS application proved its usefulness in quantitatively evaluating the subcellular distribution of lanthanum. The resulting images at the La M_5_ M_4_ edges are shown in Figure 3H; High concentrations of La (expressed in relative % atomic with C, N, and O elements) are measured close to the endothelium in luminal space while a gradient with decreasing concentrations is measured toward the plasma membrane to the center of the endothelium. Those results illustrate the chemical background reactivity of the lanthanum with cellular matrix and prove a better affinity for membrane/luminal components assumed to be glycocalyx. The last approach of this study, which consisted of the glycocalyx distribution in a volume fraction of artery, was provided by 3D (x, y, z) tomography FIB-SEM. A slice of view was acquired for each z position, as we moved along the transversal axis of the artery (Figure 4A, Supplementary Data Video S1). Results show the dynamic state of glycocalyx; some areas have no coating (Z = 0) whereas a few nanometres further (Z = +300 nm), the glycocalyx appears to be fully covering the endothelium (yellow head arrow). In addition, few intracytoplasmic vesicles with a dense white marked content are observed and some of them closed to the apical side seem to fuse with the plasma membrane and to expose their content at the luminal side (white head arrow).

### Impact of IR-CPB on Glycocalyx Morphology

**Figure 5:**
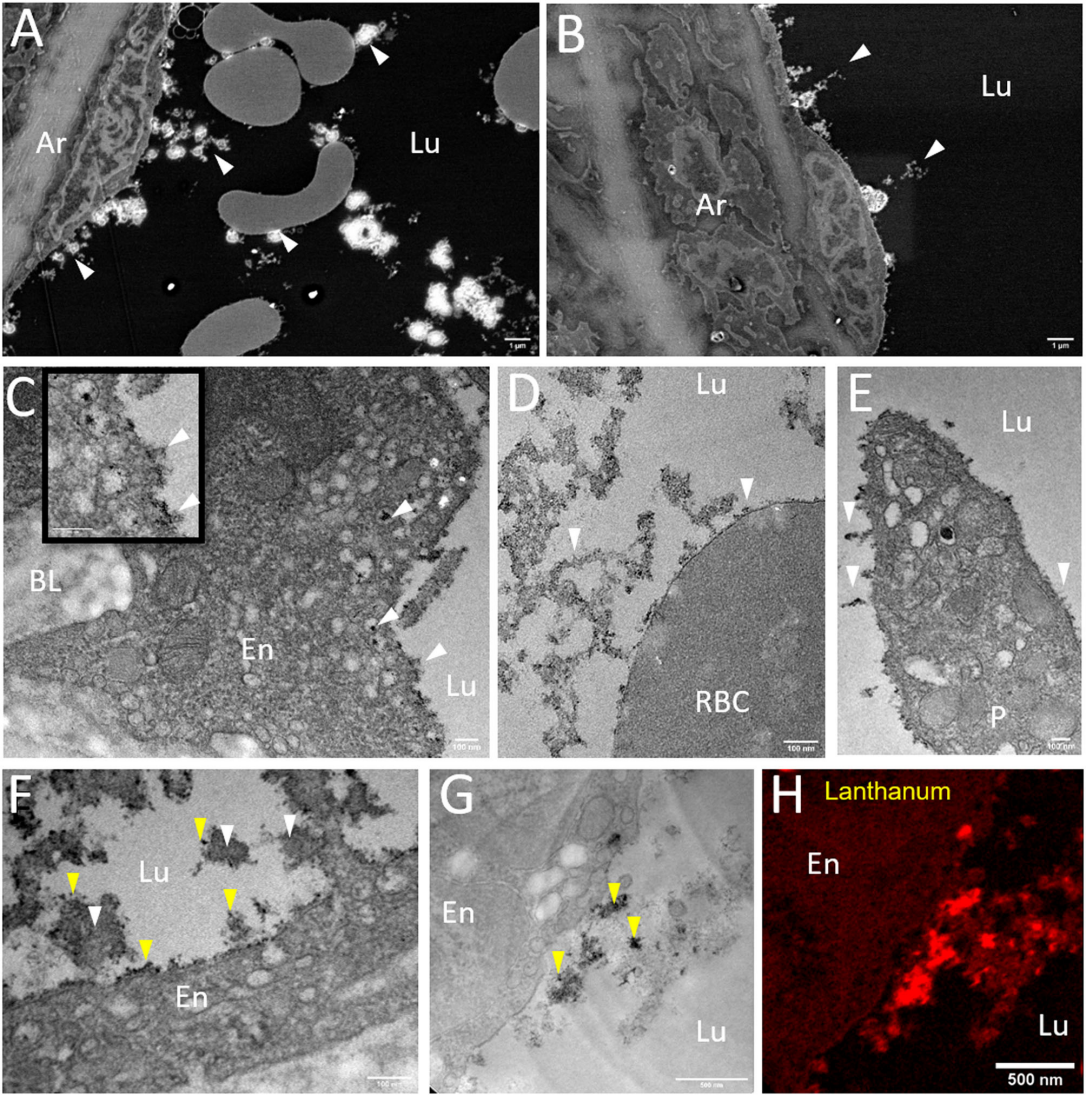
Morphology of the glycocalyx under IR-CPB conditions imaged by EM combined techniques. **(A,B)** Overview micrographs acquired by SEM observations on resin embedded ultrathin sections (scale bar: 1 μm). **(C–F)** Higher resolution TEM images showing the glycocalyx weakly developed at the endothelium, red blood and platelet surface (scale bar = 100 nm). **(F)** Presence of large components (white arrows) in luminal space reacting with lanthanum salt (yellow arrows) as shown by EFTEM imaging **(G,H)** scale bar = 0.5 μm. Ar, Artery; BL, Basal Lamina; En, Endothelium; Lu, Lumen; P, Platelet; RBC, Red Blood Cell.

**Figure 6:**
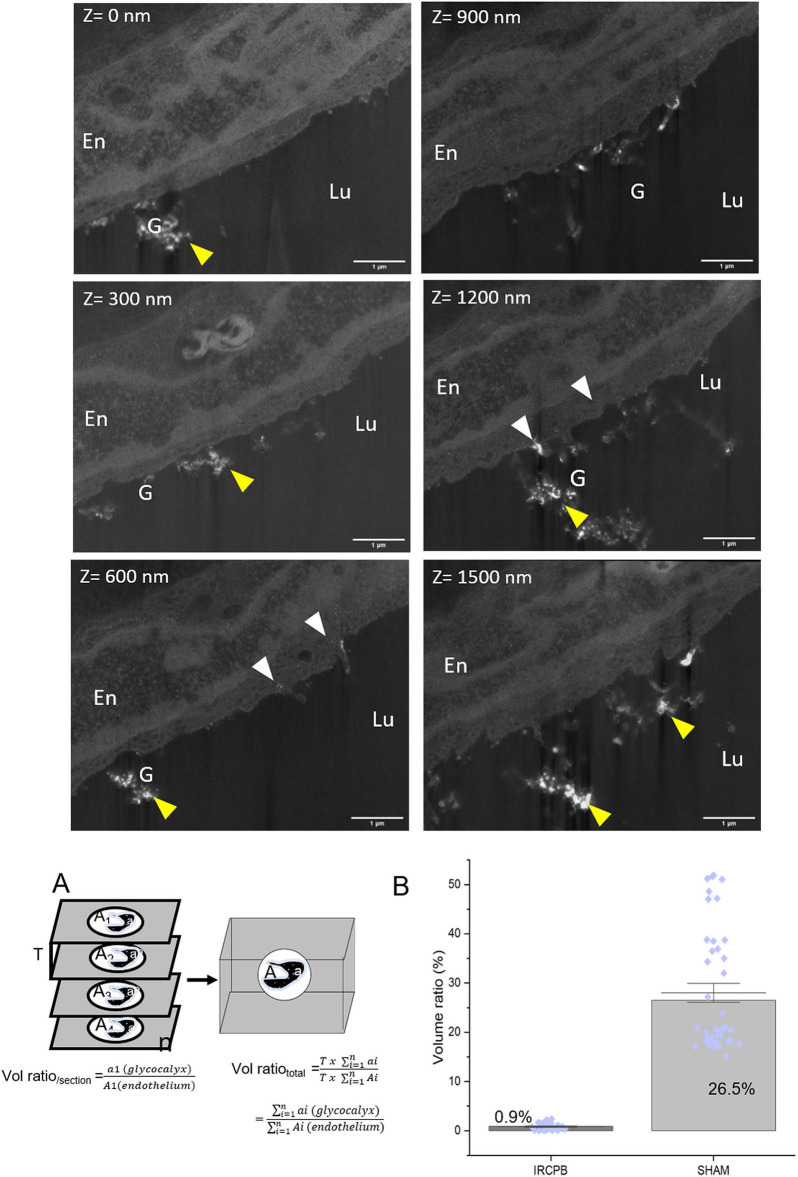
Tomography 3D FIB-SEM under IR-CPB conditions: Micrographs extracted from FIB-SEM stack at different z-position. Stack images were acquired from 30 ≪ slice and view ≫ images with a step of 50 nm. Glycocalyx (G) (yellow arrows) appears as white material at the endothelial surface (En). White arrows point endothelial vesicles filled with glycocalyx. Note the low density of glycocalyx at the endothelial surface; scale bar = 1μm. Quantitative estimation of glycocalyx volume was done using the Cavalieri method schematized in **(A)**; Data are normalized with endothelium volume. Bar graph **(B)** represents the glycocalyx volume ratio when the whole 3D tomogram is considered, and blue dots illustrate the dispersion of the glycocalyx volume ratio for each FIB-SEM-section (*n* = 31 and 40, respectively for IR-CPB and sham).

During lung transplantation, IR induced by sequential pulmonary artery clamping/de-clamping is known to cause endothelial dysfunction linked to hemostasis disorder. The routine use of CPB during the surgical procedure is controversial mainly due to its unclear contribution to endothelial dysfunction. A recent study (9) showed that CPB associated with IR further impaired endothelial function. Exploration by combined EM approaches (Figures 5A,B) reveals first a less regular distribution of glycocalyx at the endothelial surface as in the physiological state, exhibiting a cell coat more packed (Figures 5A,B). In addition, we notice a great abundance of detached/secreted glycocalyx, free in the artery lumen. At higher TEM magnification, only the membrane fraction is preserved (Figure 5C). On the apical side, in contrast to normal conditions, we noticed a large number of intracytoplasmic vesicles, of which several present a dense electron material, assumed to be glycocalyx molecules (Figure 5C). Furthermore, no lanthanum label is revealed in the basal lamina. Focusing on blood cells, the platelet shows a light superficial cell coat as well as red blood cells (Figures 5D,E). Three-dimension (3D) tomography (Figure 6, Supplementary Data Video S2) confirms the weak distribution of glycocalyx at the cell surface as well as high quantities of intracytoplasmic vesicles, on more than 1.2 μm of depth. As shown in the graphic Figure 6, the volume ratio for glycocalyx estimated by the Cavalieri method is reduced under IR CPB conditions (0.9%) compared to the normal condition (26%). In addition, no vesicle appears densely filled with glycocalyx material as observed in the physiological tomogram (Figure 4).

To prevent CPB-related endothelial injuries, one strategy is to use a priming solution, mainly to preserve the osmotic pressure. In this study, 4% gelofusine was used as a prime solution and its impact was evaluated on glycocalyx morphology. We observed the presence of large components in the luminal space that is dark dot-marked by lanthanum (Figure 5D) in a sparse manner. These components seem to interact with the endothelial membrane and glycocalyx as observed in Figure 5F. Glycocalyx was detected at the outer foils of the plasmalemma, with sometimes this characteristic “hairy” appearance and seems to form a complex with large components present in the lumen. Interaction between lanthanum and components is verified by energy loss filtered images (Figure 5H) showing localized spots of lanthanum in the components and closed to the endothelium.

## Discussion

### Combined EM Techniques: Strength and Limits

Addressing the study of the glycocalyx statically by considering a fixed and regular ultrastructure is a challenging approach requiring the improvement of suitable imaging techniques. Indeed, being in permanent contact with the blood flow, the morphology of the glycocalyx is continuously impacted by changes in the composition of plasma elements and flow variations (15), and with a balance between shedding due to shear stress and renewed synthesis (16, 17). Compared to the recent techniques such as the non-invasive sidestream dark-field imaging, or fluorescent microscopies, EM techniques are unavoidable to get information at the subcellular level despite its main drawback linked to the sample preparation procedures. Taken alone, conventional TEM gives a two-dimensional (2D) “snapshot” of a micrometric small sample (70 nm thickness) extracted from centimetric 3D features of vascular tissues, its main difficulty is to ensure its representation of the real state. Here, we propose a correlative approach by combining multi-scale EM techniques to correlate both tomographic information and ultrastructural details, of large vascular vessels which represents an advantage to estimate the “health” state of the glycocalyx. We demonstrate that single conventional TEM fixation, dehydration, and embedding in the resin can be successfully applied to focused ion beam milling, allowing us to follow the vessel surface in a volume closed to 100 μm^3^. Milling techniques promote the collection of serial images in an automated mode, a powerful alternative to serial sectioning using ultramicrotomy, and allow larger volumes of analysis than TEM tomography. Despite the optimized conditions (low beam current and platinum protective layer), long-time milling induced rapidly vertical stripes, sample drift, and charge accumulation avoiding the correct imaging of a very large volume. Sufficient contrast of glycocalyx was obtained by the addition of heavy metals (lanthanum) in the fixative solutions detectable in SEM by backscattered electrons and in TEM through elastic and inelastic electrons with chemical specificity. The introduction of lanthanum as a probe or stain in EM is not new (18). Lanthanum is a trivalent cation of high atomic number, finding its usefulness in biology as a tracer of barrier permeability and marker of cellular structure because of its reaction with molecules (19). The sulfated groups, present in the linear chain of HS, react with lanthanum allowing the tracking of proteoglycans through the cell. Lanthanum is not the only possible way to mark the glycocalyx; other positive staining markers of glycocalyx are referred in the literature. Ruthenium dyes (red ruthenium) are mainly used to stain the surface layer of cells, by binding electrostatically to the acidic mucopolysaccharides, with a higher affinity for the chondroitin/HS or hyaluronic acid. Associated with the OsO_4_, the ruthenium forms RuO_4_ which can react with various cellular compounds lipids, proteins, and oligosaccharides, and can act not only as a stain but also as a fixative. Like lanthanum, red ruthenium has an atomic number sufficiently heavy (Z = 44) to label the cell surface coat as a dense electron layer in TEM. To detect glycocalyx, it is also possible to use colloidal gold combined with wheat germ agglutinate (WGA) as proposed in the study of Baldwin et al. (20). WGA is a lectin that binds specifically to N-Acetylglucosamine residues contained in HS. Lectins coupled to gold particles are valuable tools for the localization of sugar, allowing their use in pre-or post-embedding. The main drawback of this approach results often in a low label density due to: (i) the steric hindrance induced by the particle size which limits the accessibility of gold complex to the binding site and (ii) the binding conditions dependent on the ionic strength and pH. As an alternative to these markers, tannic acid (TA) has been occasionally used in some procedures (5, 21). TA can react with diverse cellular components (proteins, membranes, and carbohydrates) but display a higher affinity for tissues rich in carbohydrates. Usually employed to improve fixation with glutaraldehyde, TA acts as a mordant by forming a complex with a metal salt such as osmium or uranyl acetate (22). Due to its inability to penetrate living or fixed plasma membrane, TA promotes the visualization of the extracellular space including glycocalyx. One of its main limitations is excessive precipitation which may occur at non-optimized concentrations. In this study, we selected lanthanum as glycocalyx marker for the following advantages: Lanthanum has a high atomic number allowing electron-scattering; Lanthanum is available under colloid or ionic forms pH dependent. The colloid form is impermeable to the cell membrane, staining the extracellular space, while ionic form (La^3+^) acts as an intracellular marker, particularly pertinent to get information on cell permeability when used before or during fixation. In addition, due to its cationic form, lanthanum may act as a calcium probe by binding to the same sites of Ca^2+^ and is well adapted to study cardiac muscle. Regarding EELS application, the loss edges energies for lanthanum are 99 eV (La N_4, 5_) and 832–849 eV (La M_5_, M_4_) and may be easily detected by the spectrometer. In contrast, Gold (Au) and Ruthenium (Ru) have no suitable edges energies: some of them are above 2,000 eV tending to get a noisy signal (Au-M_5_, M_4_ at 2,206–2,291 eV or Ru-L_3_L_2_ at 2,800–2,967 eV) or overlap with carbon signal (Ru-M_4, 5_ at 279 eV) which impaired their detection and quantification accurately.

Because of vessel diameter, studying the glycocalyx of a large artery is more challenging than of capillaries, due mostly to the pressure-flow depletion achieved when sampling, leading to brutal modification of the glycocalyx microenvironment. These effects must be added to the well-known artifact related to the EM preparation procedure. Ebong et al. (23) demonstrated the benefits of rapid freeze substitution on the preservation of the glycocalyx and outlined the importance of maintaining the adsorbed proteins and the microenvironment in the vicinity of the glycocalyx. High-pressure freezing/ultrarapid freezing contributes to immobilizing the structure near its native state where the slow reaction of aldehydes fixatives is lacking and is less effective on polysaccharides. The use of solvents during the dehydration step is an additional factor to disturb the microenvironment. Soluble components such as proteins, proteoglycans, or other molecules originating from the endothelium or the bloodstream described to preserve the charge selectivity of the permeability barrier, may contribute to the structural organization of the glycocalyx (1) by maintaining electrostatic interactions or crosslinking. Recently, Hempel et al. (24) reported the collapse of glycocalyx during the dehydration step, whatever the fixation method. Aware of these limitations, we outline the importance to acquire multiscale images. Results obtained in SEM mode on ultrathin sections produce a quality of images similar to TEM at low magnification and facilitation of large field of view informing on: (i) the luminal content in particular blood cells (density, nature, and aggregative state) their interaction with endothelium surface layer, the abundance of shed proteoglycans and (ii) the distribution of glycocalyx and precursors at the outer surface of endothelium and circulating cells as well as within intracytoplasmic vesicles. Taken together, SEM/FIB-SEM and TEM applied on the same sample prepared routinely may cover a larger explorative field of the glycocalyx and may be a powerful tool for cardiovascular research.

### Glycocalyx Morphology: Toward Criteria to Define Glycocalyx Integrity

The volume analysis supported in this study demonstrates that: (1) areas of the endothelium are densely covered by glycocalyx while others are not, under normal physiology; (2) the presence of proteoglycans in intracellular vesicles, in lamina basal, at the surface of blood circulating cells; and (3) the interaction with plasma proteins. Some hypotheses emerge from these observations. First, according to many authors (1, 6, 15–17), the glycocalyx is a dynamic structure with important turn-over. Because of their dual composition consisting of polysaccharides with a protein core, the synthesis of proteoglycans is mediated by the endoplasmic reticulum and Golgi apparatus. The newly formed products are transported to the plasma membrane by vesicular transport. On the basal side, the endothelium is separated from elastic limiting by the basement membrane constituted by lamina lucida and densa providing key functions to support cell architecture, resistance to mechanical stress, and acting as a molecular filter. Leblond and Inoue (25) demonstrated their structural organization as a three-dimensional network composed of HS proteoglycans firmly interconnected with laminin and collagen, explaining the reactivity of lanthanum with the basal lamina. Platelets cell coat was first described by Behnke et al. (26) as negatively charged mucopolysaccharides highly reactive with ruthenium or thorotrast, corroborating our observations. Cell surface proteoglycans are postulated to bind numerous ligands and macromolecules and may play a significant role in the adhesion and aggregation of platelets during hemostasis.

Furthermore, our observations suggest that IR-CPB induces a large depletion area of glycocalyx at the outer surface of the endothelium. Previous work (9) on the same model, revealed an increase of blood syndecan-1 level when IR was associated with CPB, supporting the hypothesis of glycocalyx degradation. In addition, we noticed an increased number of vesicles at the apical side, suggesting a compensatory mechanism by which the endothelium tends to restore the pool of proteoglycans. In addition, we observed large molecules reacting with lanthanum and the endothelium surface at the luminal side, which is supposed to be related to the prime solution used during CPB. It could exist a direct metal affinity of these molecules with lanthanum ions, and thus no role of the glycocalyx, or direct interaction of these molecules with the glycocalyx stained by lanthanum. It is now well established that, under physiological conditions, serum proteins and the glycocalyx interact together to stabilize the tridimensional structure of the glycocalyx in the artery lumen (27), forming the so-called endothelial surface layer by Pries et al. (28) and having a key role in the selective permeability (1). Regarding our results in IR-CPB conditions, we suggest that free or anchored glycocalyx interacts with the molecules of the prime solution. This creates a layer at the endothelium surface which may modify its environment (charge and permeability) and affect its functionality, highlighting the choice of prime solution, as a key parameter.

The combination of multiscale EM techniques contributes to the overview of the interplay between the glycocalyx, the endothelium ultrastructure, and blood circulating elements. According to our results, we propose to consider new criteria in addition to thickness to evaluate glycocalyx integrity: (i) its density at the luminal side, (ii) its interaction with circulating blood cells and proteins, (iii) the density of intracytoplasmic vesicles, and (iv) the basal lamina organization.

## Conclusion

The present EM multiscale methodology has shown major advantages over TEM in vascular glycocalyx research. The first advantage comes from the possibility to image ultrathin resin embedded sections issued from the same sample both by SEM and TEM at a different scale. This allows to cover a large field (μm) then high resolution (nm) imaging and to acquire both ultrastructural (TEM) and chemical information, opening new insights with the EELS. The second advantage, offered by the tomography by milling on block face (FIB-SEM), is to obtained data in a volume higher than TEM tomography. From a single standard sample preparation procedure, this methodology could improve glycocalyx morphology studies and provides new insights about the dynamics and biological role of the glycocalyx as well as its evolution in disease. As previously described, some limitations still exist and further investigations are needed to deepen and optimized this new imaging approach to be used routinely.

## Data Availability Statement

The original contributions presented in the study are included in the article/Supplementary Material, further inquiries can be directed to the corresponding author.

## Ethics Statement

The animal study was reviewed and approved by the French Ministère de l'Enseignement Supérieur et de la Recherche Direction Générale pour la Recherche et l'Innovation (No APAFIS 2016102718162386-V2).

## Author Contributions

LC: TEM/SEM investigations, analysis, and writing the original manuscript. JS and JB: cardiovascular investigations, analysis, and surgical procedure. CC: TEM instrument manager. FC: FIB-SEM tomography. VR and PP: team management. JM-B: cardiovascular investigations, analysis, and correcting the original manuscript. All the authors contributed to the article and approved the submitted version of the manuscript.

## Funding

This study was funded by the European Union with European Regional Development Fund (FEDER) and by Région Normandie in the framework project CellSTEM.

## Conflict of Interest

The authors declare that the research was conducted in the absence of any commercial or financial relationships that could be construed as a potential conflict of interest.

## Publisher's Note

All claims expressed in this article are solely those of the authors and do not necessarily represent those of their affiliated organizations, or those of the publisher, the editors and the reviewers. Any product that may be evaluated in this article, or claim that may be made by its manufacturer, is not guaranteed or endorsed by the publisher.

## Supplementary Material

Supplementary Video 1 Tomography 3D FIB-SEM showing the evolution of the glycocalyx (white dense material) in a volume fraction of a lung artery under physiological conditions. Scale bar = 1 μm.

Supplementary Video 2 Tomography 3D FIB-SEM showing the evolution of the glycocalyx (white dense material) in a volume fraction of a lung artery under IR-CPB conditions. Scale bar = 1 μm. IR, ischemia-reperfusion; CPB, cardiopulmonary bypass.
